# Genome-Wide Profiling of p63 DNA–Binding Sites Identifies an Element that Regulates Gene Expression during Limb Development in the 7q21 SHFM1 Locus

**DOI:** 10.1371/journal.pgen.1001065

**Published:** 2010-08-19

**Authors:** Evelyn N. Kouwenhoven, Simon J. van Heeringen, Juan J. Tena, Martin Oti, Bas E. Dutilh, M. Eva Alonso, Elisa de la Calle-Mustienes, Leonie Smeenk, Tuula Rinne, Lilian Parsaulian, Emine Bolat, Rasa Jurgelenaite, Martijn A. Huynen, Alexander Hoischen, Joris A. Veltman, Han G. Brunner, Tony Roscioli, Emily Oates, Meredith Wilson, Miguel Manzanares, José Luis Gómez-Skarmeta, Hendrik G. Stunnenberg, Marion Lohrum, Hans van Bokhoven, Huiqing Zhou

**Affiliations:** 1 Department of Human Genetics, Nijmegen Centre for Molecular Life Sciences, Radboud University Nijmegen Medical Centre, Nijmegen, The Netherlands; 2 Department of Molecular Biology, Faculty of Science, Nijmegen Centre for Molecular Life Sciences, Radboud University Nijmegen, Nijmegen, The Netherlands; 3 Centro Andaluz de Biología del Desarrollo, Universidad Pablo de Olavide, Consejo Superior de Investigaciones Científicas, Sevilla, Spain; 4 Centre for Molecular and Biomolecular Informatics, Nijmegen Centre for Molecular Life Sciences, Radboud University Nijmegen Medical Centre, Nijmegen, The Netherlands; 5 Fundación Centro Nacional de Investigaciones Cardiovasculares Carlos III, Madrid, Spain; 6 Department of Clinical Genetics, Children's Hospital at Westmead, Westmead, Australia; 7 Department of Cognitive Neuroscience, Donders Institute for Brain, Cognition, and Behavior, Radboud University Nijmegen Medical Centre, Nijmegen, The Netherlands; The University of North Carolina at Chapel Hill, United States of America

## Abstract

Heterozygous mutations in *p63* are associated with split hand/foot malformations (SHFM), orofacial clefting, and ectodermal abnormalities. Elucidation of the p63 gene network that includes target genes and regulatory elements may reveal new genes for other malformation disorders. We performed genome-wide DNA–binding profiling by chromatin immunoprecipitation (ChIP), followed by deep sequencing (ChIP–seq) in primary human keratinocytes, and identified potential target genes and regulatory elements controlled by p63. We show that p63 binds to an enhancer element in the SHFM1 locus on chromosome 7q and that this element controls expression of *DLX6* and possibly *DLX5*, both of which are important for limb development. A unique micro-deletion including this enhancer element, but not the *DLX5/DLX6* genes, was identified in a patient with SHFM. Our study strongly indicates disruption of a non-coding cis-regulatory element located more than 250 kb from the *DLX5/DLX6* genes as a novel disease mechanism in SHFM1. These data provide a proof-of-concept that the catalogue of p63 binding sites identified in this study may be of relevance to the studies of SHFM and other congenital malformations that resemble the p63-associated phenotypes.

## Author Summary

Mammalian embryonic development requires precise control of gene expression in the right place at the right time. One level of control of gene expression is through cis-regulatory elements controlled by transcription factors. Deregulation of gene expression by mutations in such cis-regulatory elements has been described in developmental disorders. Heterozygous mutations in the transcription factor *p63* are found in patients with limb malformations, cleft lip/palate, and defects in skin and other epidermal appendages, through disruption of normal ectodermal development during embryogenesis. We reasoned that the identification of target genes and cis-regulatory elements controlled by p63 would provide candidate genes for defects arising from abnormally regulated ectodermal development. To test our hypothesis, we carried out a genome-wide binding site analysis and identified a large number of target genes and regulatory elements regulated by p63. We further showed that one of these regulatory elements controls expression of *DLX6* and possibly *DLX5* in the apical ectodermal ridge in the developing limbs. Loss of this element through a micro-deletion was associated with split hand foot malformation (SHFM1). The list of p63 binding sites provides a resource for the identification of mutations that cause ectodermal dysplasias and malformations in humans.

## Introduction

The p63 protein encoded by the *TP63* gene is a transcription factor of the p53 family and functions as a master regulator of ectodermal development. The key function of p63 during ectodermal development is underscored by phenotypic features in *p63* knockout mice [1], [2] and in *p63* knock-down zebrafish [3], [4]. The developmental abnormalities in animal models are reminiscent of those in *p63*-associated human disorders. Heterozygous mutations in *p63* give rise to at least seven dominantly inherited clinical conditions with three major characteristics, ectrodactyly (also known as split hand/foot malformation, SHFM), orofacial clefting and ectodermal dysplasia with defects in skin, hair, teeth, nails and exocrine glands [5], [6]. There is a clear genotype-phenotype correlation in *p63*-associated disorders [7]. The most prominent of these disorders is the Ectrodactyly Ectodermal dysplasia and Cleft lip/palate syndrome (EEC, OMIM 604292) which combines all of the three phenotypic hallmarks and is almost invariably caused by missense mutations in the DNA binding domain of *p63*. Ankyloblepharon Ectodermal defects Cleft lip/palate syndrome (AEC, OMIM 106260) is caused by mutations in the SAM domain of the p63 that is involved in protein interaction. Nonetheless, mutations of *p63* can explain only a minority of patients with only one of the three cardinal features, such as in patients with isolated SHFM (∼10%) and in patients with isolated cleft lip/palate (∼0.1%) [7]. There remains a large group of ectodermal dysplasia syndromes with phenotypes that resemble *p63*-associated syndromes [8]. The genetic basis of many of these clinically related conditions, referred to as the *p63* phenotype network, is presently unknown.

There is ample evidence that diseases clustering within such phenotype networks are caused by mutations in functionally related genes that constitute a gene network [9]–[11]. Elucidation of functional interactions among genes within the *p63* gene network, their encoded proteins and regulatory elements which control expression of these genes will therefore provide new candidate genes for genetic disorders from the *p63* phenotype network. Identifying target genes and cis-regulatory elements controlled by p63 is an important step in dissecting the *p63* gene network. Previous studies have focused on transcriptional target genes of p63 identified through individual candidate gene approaches [12]–[14] or through genome-wide approaches [15]–[19]. However, the role of regulatory elements controlled by p63 in transcription has not yet been addressed so far.

Split hand/split foot malformation (SHFM, OMIM 183600) is characterized by a deficiency of the central rays of the hands and feet, resulting in missing or malformed digits. SHFM may be isolated (non-syndromic) or be associated with other developmental anomalies (syndromic). Six distinct chromosomal loci for non-syndromic SHFM have been reported. Specific gene mutations have been identified in SHFM6 and SHFM4. SHFM6 (OMIM 225300, chromosome 12q13) is caused by a homozygous *WNT10B* mutation and it is the only autosomal recessive form of SHFM [20]. SHFM4 (OMIM 605289, chromosome 3q27) is caused by *p63* mutations [21]. Chromosomal aberrations underlie three other types of isolated SHFM: 7q21 deletions and re-arrangements in SHFM1 (OMIM 183600) [22], 10q24 duplications encompassing the *Dactylin* gene *(FBXW4)* in SHFM3 (OMIM 600095) [23], and 2q31 deletions encompassing the *HOXD* gene cluster in SHFM5 (OMIM 606708) [24], [25]. In addition, linkage analysis has mapped SHFM2 (OMIM 313350) to chromosome Xq26 [26].

The SHFM1 locus on chromosome 7q21 has been delineated by various translocations, inversions, deletions and duplications [27]. The smallest region of overlapping deletions in SHFM1 patients [28] encompasses several genes: *DYNC1I1*, *SLC25A13*, *DSS1*, *DLX5* and *DLX6*, of which only *DLX5* and *DLX6* have been shown to clearly play a role in early limb development. *Dlx5 and Dlx6* are highly expressed in the apical ectodermal ridge (AER) of the developing limbs of mice [29]–[31] and in the fins of zebrafish [3], [4]. The AER is critical for limb outgrowth and patterning [32] and there is strong evidence that a failure to maintain the AER signaling is the main pathogenic mechanism in ectrodactyly [33]. The importance of the *DLX5/DLX6* genes in limb development has been highlighted in mouse models. *Dlx5* deficient mice do not show any limb defects [30]. However, an SHFM-like phenotype has been observed when both *Dlx5* and *Dlx6* were simultaneously deleted (*Dlx5/Dlx6^−/−^*). The limb developmental phenotype in *Dlx5/Dlx6^−/−^* mice could be fully rescued by overexpression of *Dlx5* in the AER [29], [34]. These observations suggest that the *DLX5* and *DLX6* genes cooperate in limb development by controlling a common developmental program. *DLX5* and *DLX6* are further expressed in the craniofacial prominence, the otic vesicle and in the brain [29]–[31], which correlates well with the hearing loss and mental retardation that are present in 30% of the SHFM1 patients [27]. While *DLX5* and *DLX6* are obvious candidate genes for SHFM1, mutations have not been found in either of the two genes.

Here, we used a genome-wide DNA-binding profiling approach using Chromatin Immunoprecipitation (ChIP) followed by deep sequencing (ChIP-seq) in human primary keratinocytes to generate a catalogue of highly informative target genes and regulatory elements controlled by p63. One cis-regulatory element identified by DNA-binding profiling is located in the SHFM1 critical region and acts as an enhancer element for gene expression mediated by p63 during embryonic limb development. Our data indicate that loss of this element leads to SHFM1. This example illustrates that our catalogue of p63 binding sites can identify candidate genes and loci for the elucidation of disorders from the p63 phenotype network.

## Results

### Genome-wide p63 binding profile in human primary keratinocytes

**Figure 1:**
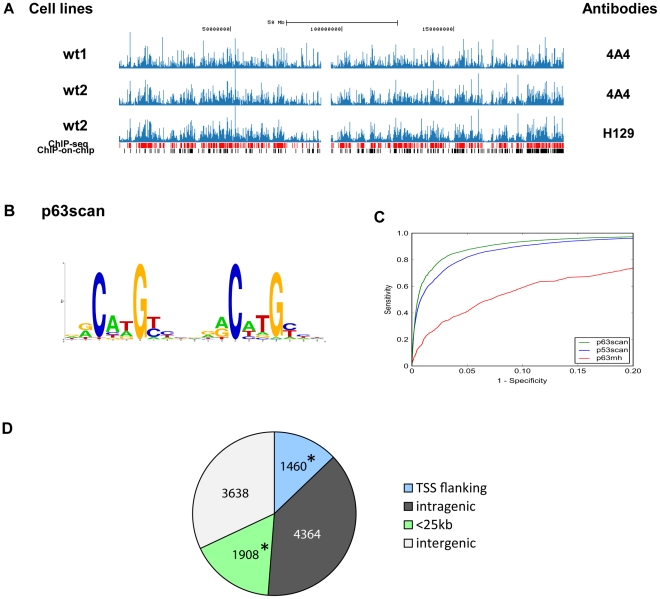
Characterization of identified p63 binding sites. (A) A screenshot of chromosome 3 using UCSC genome browser shows similar DNA-binding profiles from our ChIP-seq analysis of two normal human primary keratinocyte cell lines (wt1 and wt2) with two different antibodies (4A4, pan-p63 and H129, α-specific). The p63 binding sites analyzed with MACS [35] using *P* value 1×10^−9^ are shown in red, and previously reported p63 binding sites in ChIP-on-chip analysis [17] are shown in black. (B) The p63 motif was identified by a *de novo* motif analysis (see Materials and Methods). Based on a previously developed p53scan algorithm [37] with this newly identified p63 Positional Weight Matrix (PWM), p63scan was developed. (C) The performance of p63scan using the *de novo* identified motif shows superior sensitivity compared to previously reported p63MH without compromising specificity. (D) Distribution of the p63-binding site location relative to RefSeq genes. Locations of binding sites are divided into: TSS flanking region (5 kb upstream of TSS, first exon and first intron), intragenic region (all introns and exons except first), <25 kb (5–25 kb upstream or 25 kb downstream of last exon), or intergenic regions (everything else). The asterisk represents significant enrichment.

The most common isoform of p63, ΔNp63α, is highly expressed in the basal layer of the epidermis that consists mainly of keratinocytes. We therefore established human primary keratinocyte cultures (HKCs) from adult skin as our model system to elucidate *p63* gene networks under physiological conditions. To identify target genes and regulatory elements controlled by p63, high-resolution global binding profiles of p63 were obtained from HKC cell lines established from two unrelated control individuals (wt1 and wt2) by ChIP-seq analysis using two antibodies recognizing different epitopes in p63 (4A4 and H129). Analysis of the sequenced reads using the peak recognition algorithm of Model-based Analysis of ChIP-Seq (MACS) [35] gave a highly significant overlap of 11,369 peaks from three profiles (*P*<1E-300) (Figure 1A). Overlapping peaks were therefore considered as a collection of high-fidelity p63 binding sites in HKCs. Indeed, a set of 17 representative binding sites of various peak heights, conservation scores and consensus motif scores (see below) were tested with independent ChIP followed by qPCR analysis (ChIP-qPCR) with two antibodies (4A4 and H129) and all of them could be validated (Table S1, Figure S1). This confirmed that the obtained p63 binding profile is highly reliable.

To determine the specific p63-binding sequences in the detected binding sites, a *de novo* consensus motif prediction pipeline was applied to generate a Position Weight Matrix (PWM) (see Materials and Methods for details). A highly significant consensus sequence was identified that is similar to the previously reported p63 and p53 consensus motifs (*P*<1E-250) [17], [18], [36], [37] (Figure 1B, Table S2). An additional significant AP1-like motif that can be bound by c-Fos and c-Jun proteins [38] was also identified in the detected binding sites (*P*<1E-50, Table S2). We combined our previously developed p53scan algorithm [37] with the newly identified p63 PWM, hereafter referred to as p63scan. Comparison of p63scan with the previously described motif algorithms p63MH [36] and p53scan [37] showed that p63scan had clearly higher sensitivity for motif recognition without compromising the specificity (Figure 1C). A slight increase of motif scores correlated with an increase of peak heights of the binding sites (Figure S2), suggesting a stronger binding of p63 to binding sites with higher motif scores. Using p63scan, 10,702 out of a total 11,369 p63 binding sites (94%) were found to contain at least one p63 motif (motif score 4.74, False Discovery Rate, FDR 10%). The high percentage of motif-containing binding sites indicates that most binding sites identified in this study participate in direct binding of p63. The *de novo* consensus motif prediction pipeline was applied to the subgroup of binding sites without p63 binding motifs to search for consensus motifs of other transcription factors or novel binding motifs of p63. A degenerate p63 binding motif was identified, and interestingly, the AP1 motif was also found more strongly enriched as compared to the motif analysis of all binding sites (Table S2). An alternative approach also was taken to examine all known motifs of transcription factors in the TRANSFAC Professional database (version 2009.3) [39] for their significant over-representation (*P*<1E-10, after Bonferroni correction) in the p63 motif-less binding sites relative to the p63 motif-containing sites. Consistent with the *de novo* consensus motif search, the AP1 motif as well as the BACH1 and BACH2 motifs that are similar to AP1 was found (Table S3). These data suggest that p63 can bind to DNA by collaborating with other transcription factors such as c-Jun or c-Fos. Interestingly, a previous report showed that p63 binds to an AP1 responsive element to regulate Keratin 1 in keratinocytes in a c-Jun dependent manner [40]. No other novel consensus binding motifs were detected.

Out of 11,369 binding sites, 1460 lie between 5 kb upstream of the transcription start site (TSS) and the end of the first intron of genes, referred to as TSS flanking regions, and 1908 binding sites are located within 25 kb distance to a gene (<25kb region) (Figure 1D). Statistical analysis showed that binding sites at these two chromosomal regions are enriched compared to genomic distributions of all binding sites (*P*<0.001). Genomic distribution of binding sites with or without a p63 binding motif was similar to that of all binding sites (Figure S3). In total, 10,895 genes had one or more p63 binding sites within 25 kb up- and down-stream of the gene, and they were considered as potential target genes (PTGs) of p63. GO annotation of these 10,895 PTGs using DAVID Bioinformatic Resources 6.7 (NIAID, NIH) [41] showed a non-random distribution with enrichment in functional categories of biological processes, such as development, adhesion, cell communication and intracellular signaling cascade (Table S4). Binding sites with or without a p63 motif were also mapped to genes as separate subgroups, and 10,438 and 944 genes, respectively, have p63 binding sites within 25 kb of the gene. GO annotation of genes mapped by binding sites with motifs resulted in very similar GO terms as annotation of all PTGs (Table S2). However, 944 genes mapped by binding sites without motifs were seemingly involved in slightly different biological processes (Table S4).

**Figure 2:**
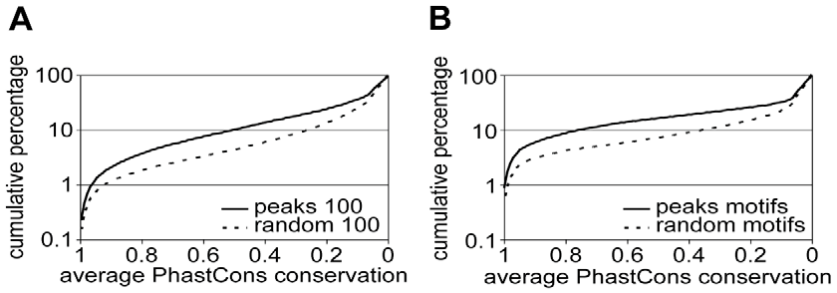
Conservation of p63 binding sites and motifs in vertebrates. The percentage of p63 binding sites (y-axis) is plotted against decreasing cut-off values of the PhastCons Conservation Score (PCCS) (x-axis). A point on the line indicates the percentage of binding sites with that particular PCCS or higher. (A) Average PCS of 100-nucleotide regions centered at the summit of 11,425 p63 binding peaks (peaks 100) and that of 100,000 100nt regions randomly chosen from the whole genome (random 100). (B) Average PCCS of 10,659 10-nt p63 motifs detected by p63scan in the 11,425 p63 binding peaks (peaks motifs) and of the 7,600 motifs detected in 100,000 random genomic regions (random motifs).

The *p63* gene arose from two sequential gene duplications at the root of the vertebrates and has unambiguous orthologues only in that taxon [42]. We therefore assessed the evolutionary conservation of the identified binding sites and the p63 consensus motifs therein in aligned vertebrate genomes (PhastCons). The identified binding sites had higher average PhastCons Conservation Scores (PCCS) and were significantly more conserved than random sequences of the same size (Figure 2A). Moreover, PCCS of motifs identified in the p63 binding sites were also compared to that in the random genomic regions. By p63scan, 10,702 motifs were identified in 11,369 p63 binding sites and they were more conserved than 4,003 motifs identified in 100,000 random genomic regions (Figure 2B). These data support the functionality of the identified p63 binding sites. We did not observe a correlation between PCCS and peak height (Figure S4A) or a clear difference in PCCS of binding sites with and without p63 binding motifs (Figure S4B).

### Association of potential p63 target genes with the disease phenotypes

To validate whether the identified binding sites represented target genes and regulatory elements relevant to the *p63*-associated and other diseases with clinical similarities, the OMIM database was searched for diseases associated with the 10,895 potential target genes in this study. We found 904 OMIM disease entries associated with these genes (Table 1, Table S5), referred to as p63 potential target gene-associated diseases (PTG-associated diseases). To assess the relationship amongst PTG-associated diseases, their clinical features were analysed by text mining (Table S6) and evaluated with a similarity algorithm [43] (Table 1). The potential target genes of p63 do not have strong tendency to associate with diseases (*P* = 1). However, the feature terms in PTG-associated diseases are similar, as the similarity score of these diseases (0.284) is significantly higher than for a random distribution (0.200) (*P*<1E-6). This shows that PTGs are associated with diseases that have similar clinical phenotypes. Features associated with p63 syndromes are enriched in the top 10% of overrepresented feature terms for the PTG-associated syndromes (*P*<1E-28). Many of these terms such as stem cell and epithelium reflect p63 functioning (Table S6). This suggests that identified PTGs tend to cause similar disease phenotypes to p63-associated diseases. We did not observe a significant difference between terms derived from motif-containing binding sites or those from motif-less binding sites (Table S6). The significant similarity of disease features of PTGs suggests that these binding sites are relevant to p63-related developmental disorders.

**Table 1:** Diseases associated with genes containing p63 binding sites.

	Diseases associated with genes with p63 binding sites	Total diseases in OMIM associated with genes	*P* value^C^
No. of diseases	904	2033	1
Similarity score of feature terms^A^	0.284	0.200^B^	<10^−6^

A Text mined feature terms that are associated with diseases are calculated for their similarity scores (0, no overlap and 1, identical overlap).

B Randomized sampling of 904 diseases from OMIM database are used for the similarity score.

C Chi-square test.

To assess whether p63 binding sites can function as regulatory elements in the p63-related disease network, we focused on SHFM. From the human malformation disease database POSSUM [44] and the Jackson Laboratory's Mouse Genome Database [45], 20 genes were selected based on their localization in the human SHFM loci (Table S7). In addition, these genes are known either to associate with SHFM in human or to have similar phenotypes in mice. These genes are further referred to as SHFM-associated genes (Table S7). As regulatory elements can function over a large distance but might be blocked by insulator elements that are defined by CTCF binding sites [46], p63 binding sites were searched in broad chromosomal regions containing the SHFM-associated genes (up to 300kb from the genes) provided that no known CTCF binding sites are located between the binding site and the gene. With these criteria, p63 binding sites were identified near 12 SHFM-associated genes (Table 2). We propose that these p63 binding sites are potential regulatory elements that might contribute to SHFM.

**Table 2:** p63-binding sites in the chromosomal regions near SHFM-associated genes (hg 18).

Gene	Chr	No. of peaks*	Location of peaks
p63	chr3	4	191025109–191025933; 191034090–191035150; 191100798–191101709; 191150499–191151202
SNX3	chr6	3	108392374–108393394; 108590949–108591991; 108862963–108863753
GJA1	chr6	1	121848018–121848681
FOXP2	chr7	6	113725067–113725994; 113840901–113841821; 114182940–114183839; 114211827–114212755; 114250288–114251274; 114344204–114345050
DSS1	chr7	8	96029986–96030688; 96079266–96079792; 96110917–96111692; 96132694–96133405; 96177547–96178220; 96194972–96195903; 96337204–96338894; 96388802–96389666
DLX6	chr7	3	96194972–96195903; 96337204–96338894; 96388802–96389666
DLX5	chr7	3	96194972–96195903; 96337204–96338894; 96388802–96389666
FBXW4	chr10	1	103514476–103515097
SMC3	chr10	4	112103092–112103960; 112147937–112149561; 112223542–112224445; 112243209–112244187
BRCA2	chr13	1	31904891–31905392
CDH3	chr16	2	67249490–67250527; 67259342–67260368
PORCN	chrX	1	48204855–48205660

*Binding sites of p63 were searched within 300kb distance to the SHFM-associated genes and without CTCF binding sites in between.

### Identification of p63 binding sites in a 7q21.3 microdeletion in an SHFM1 patient

**Figure 3:**
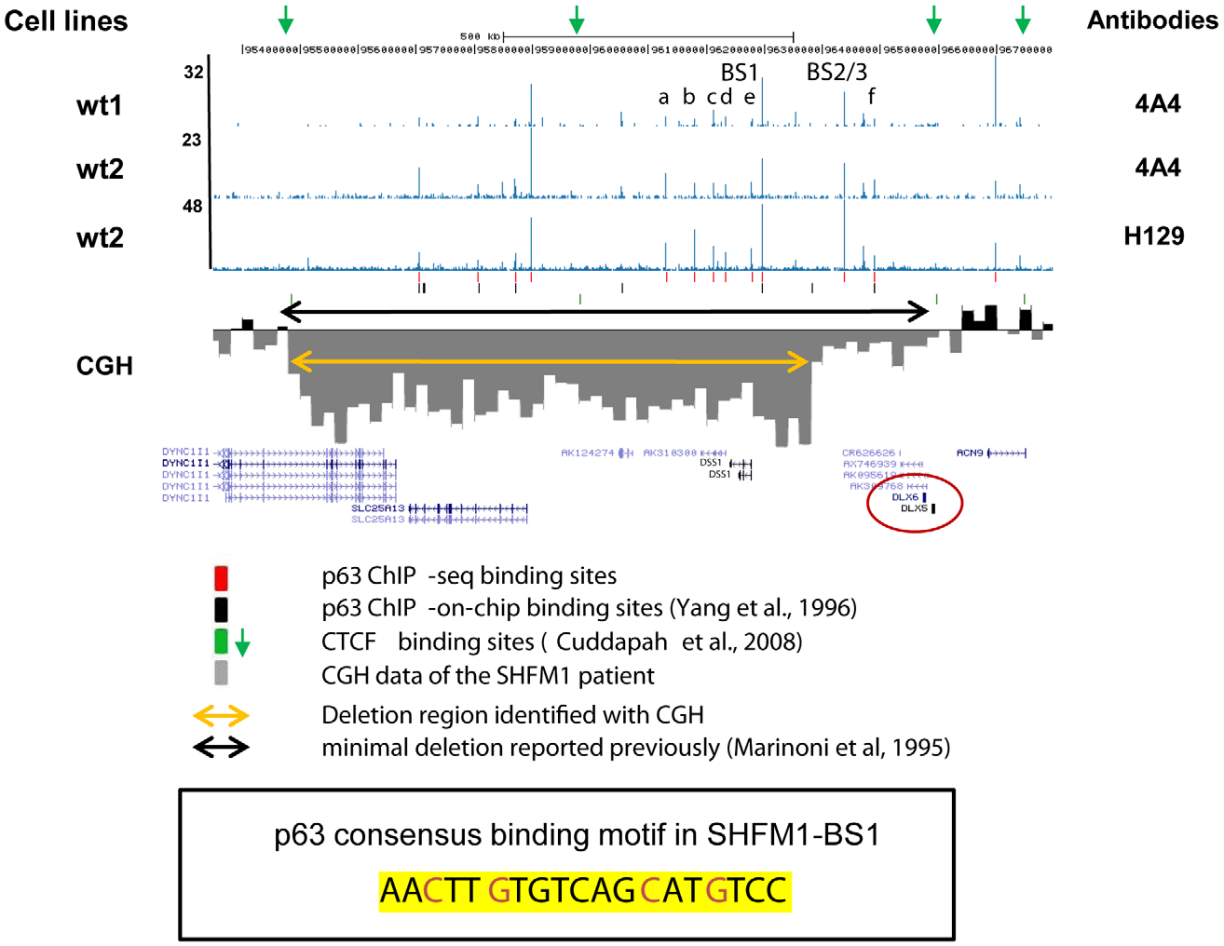
A microdeletion at SHFM1 locus on chromosome 7. A screenshot of UCSC genome browser shows the p63 binding profile from two HKC cell lines (wt1 and wt2) with two p63 antibodies (4A4 and H129) near *DLX5* and *DLX6* on chromosome 7. SHFM1-BS1, -BS2 and -BS3 are three high peaks and a–f are low ones. SHFM1-BS2 and -BS3 are in close proximity and therefore seen as a single peak in this large genomic view. Ultra-high Comparative Genomic Hybridization array analysis performed with DNA of a SHFM1 patient showed a chromosomal deletion of 880 kb on chromosome 7 (95,390,000–96,270,000, hg18, the orange arrow) which includes *DSS1*, *SLC25A13* and part of *DYNC1I1* but not *DLX5* and *DLX6*. Breakpoints and 20kb averaged log2 ratios were visualized in the genome browser. A previously reported minimal deletion determined by markers D7S527 and D7S1796 [28] is marked with a black arrow. Binding sites identified in this ChIP-seq study and in previous ChIP-on-chip [17] are labeled in red and black, respectively. CTCF binding sites revealed by previous ChIP-seq analysis [46] are labeled in green and two binding sites (chr7: 95,882,240–95,882,467 and 96,495,007–96,495,206) that define the enhancer activity of SHFM1-BS1 are marked by green arrows. A p63 consensus binding motif was identified in SHFM1-BS1 with the essential cytosine and guanine bases labeled in red.

In the SHFM1 locus on chromosome 7, several deletions have been identified which invariably contain the *DLX5* and *DLX6* genes as well as *DYNC1I1*, *SLC25A13* and *DSS1* (Figure S5) [28], [47]–[51]. We identified a new patient with non-syndromic SHFM (for clinical phenotype, see Materials and Methods) and a novel microdeletion of chromosome 7q21 by a targeted 385K chromosome 7-specific microarray. Surprisingly, the 880kb chromosomal deletion at 7q21.3 encompassed *DSS1*, *SLC25A13* and part of *DYNC1I1* but left the SHFM1 candidate genes *DLX5* and *DLX6* intact (Figure 3). The deletion was confirmed by genomic qPCR analysis (Figure S6). Compared with the previously reported minimal chromosomal deletion (Figure S5) [28], [47]–[51], the protein-coding genes in the overlapping region are *DYNC1I1*, *SLC25A13* and *DSS1* but these are not likely to contribute to the phenotype [48], [52], [53]. We therefore hypothesized that disruption of one or more regulatory elements caused the SHFM1 phenotype. To test this hypothesis, p63 binding sites were searched in the chromosomal region spanning the *DLX5/DLX6* genes, taking into account the published CTCF binding sites to define the borders of enhancer activity [46]. Consistent with our hypothesis that *DLX5/DLX6* are controlled by long distance regulatory elements, *DLX5/DLX6* are located in a broad chromosomal region between two CTCF binding sites (chr7: 95882240–95882467 and chr7: 96495007–96495206) spanning approximately 600kb (green arrows in Figure 3). This region contains nine putative p63 binding sites that were identified by our ChIP-seq analysis. These include three high peaks (SHFM1-BS1, -BS2 and -BS3) and six lower ones (a–f) (Figure 3, Table 3).

**Table 3:** PhastCons Conservation Scores (PCCS) of the identified p63 binding sites that potentially function as enhancer elements.

binding sites	start	end	PCCS*
a	96030294	96030493	0.434
b	96079364	96079563	0.234
c	96111217	96111416	0.041
d	96133015	96133214	0.010
e	96177897	96178096	0.043
BS1	96195551	96195750	0.456
BS2/BS3	96337526	96337725	0.002
f	96389201	96389400	0.088

*Average PhastCons Conservation Score of the binding sites (hg18).

### A p63 binding site within the SHFM1 deletion acts as an enhancer element in limb development

**Figure 4:**
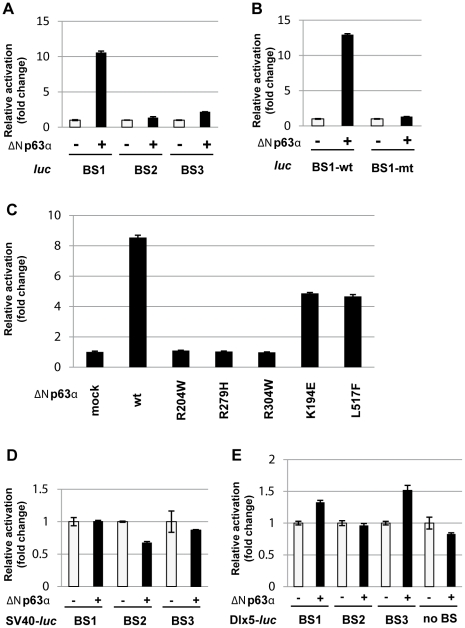
Transient transfection analysis of p63 binding sites at SHFM1 locus on chromosome 7. (A) The p63 binding sites SHFM1-BS1, -BS2 and -BS3 were tested in transient transfection assays in Saos2 cells. Transcription of the *luciferase* reporter was strongly activated by ΔNp63α through SHFM1-BS1 binding site but only weakly activated through -BS2 and -BS3. (B) Activation was abolished when point mutations were introduced into the p63 binding motif in the SHFM1-BS1 binding site where the essential cytosine and guanine bases were mutated to adenosines. (C) Activation was impaired by p63 EEC mutations R204W, R279H and R304W and slightly reduced by SHFM1 mutation K194E and AEC mutation L517F. (D) No additional activation was observed when SHFM1-BS1, -BS2 and -BS3 were cloned in front of the SV40 promoter driving *luciferase*. (E) No activation was observed on the mouse Dlx5 promoter (no BS) and when SHFM1-BS1, -BS2 and -BS3 were cloned in front of the Dlx5 promoter.

To identify the binding sites potentially important for limb development, the average PhastCons conservation score (PCCS) [54] of each of the nine binding sites was examined. We found that SHFM1-BS1 had the highest PCCS (0.456) (Table 3) that belongs to the top-ranking 11.6% of all 11,369 binding sites (Figure 2 and Figure S3). To test the functionality of p63 binding sites, the three high p63 binding peaks, SHFM1-BS1, -BS2 and -BS3, were cloned directly in front of a luciferase reporter that is followed by the SV40 enhancer to test whether they are responsive to p63 transactivation. Transient transfection assays showed that only SHFM1-BS1 was highly responsive to p63 (Figure 4A). Transactivation activity was completely abolished by mutations in the p63 binding motif present in SHFM1-BS1 (Figure 4B, motif shown in Figure 3), indicating that the observed transactivation is p63-specific. Mutations in the DNA-binding domain of *p63*, R204W, R279H and R304W, that are found in EEC syndrome disrupted transactivation, whereas mutations found in non-syndromic SHFM4, K194E, and in AEC syndrome, L517F, reduced the transactivation activity not more than 2-fold (Figure 4C). Based on the structure of the DNA-binding domain in p53 that is highly homologous to that in p63, lysine 194 (Q165 in p53) is located in the DNA-binding domain but does not have direct contact with DNA [5], [55]. The AEC syndrome mutation L517F is located in the SAM domain of p63. Therefore these mutations are unlikely to have major effect on p63 DNA-binding. To examine the enhancer activity of SHFM1-BS1, -BS2 and -BS3, these elements were cloned in front of the SV40 promoter or endogenous mouse *Dlx5* and *Dlx6* promoters that drive the *luciferase* gene, but no clear additional activation upon co-transfection of p63 was observed (Figure 4D and 4E and data not shown). Furthermore, in the absence of the enhancer, we did not detect p63 activation on the *Dlx5* promoter (Figure 4E, no BS) that was previously reported [14]. This discrepancy is probably due to different cells used in transient transfection assays. These results indicate that enhancer activity controlling expression of *DLX5* and *DLX6* genes may not be correctly recapitulated in a cellular system irrelevant to limb development.

**Figure 5:**
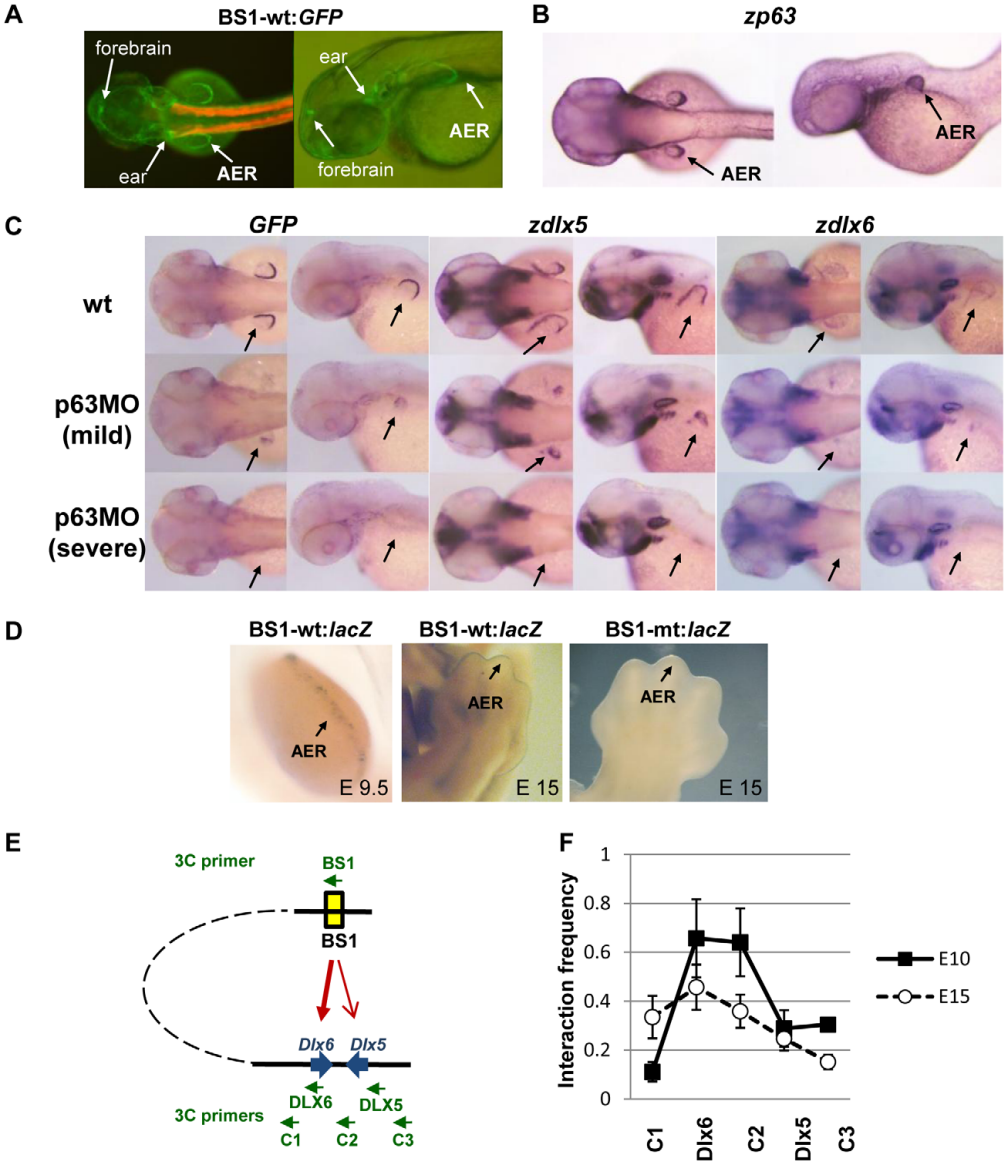
Functional analysis of SHFM1-BS1 to control gene expression in zebrafish and mice. (A) SHFM1-BS1 was cloned in a reporter construct carrying *GFP*. Expression of the *GFP* gene in zebrafish showed that SHFM1-BS1 can control gene expression at the apical ectodermal ridge (AER), the ear and the forebrain. The red fluorescence expressed in the muscles in the first zebrafish panel corresponds to the positive control of transgenesis. (B) Expression of *zp63* in the AER was detected by *in situ* hybridization. (C) Expression patterns of transgene *GFP* controlled by SHFM1-BS1, *zdlx5a* and *zdlx6a* were analysed by *in situ* hybridization in 48hpf zebrafish embryos. The same specific expression of these genes was observed in the AER. Treatment of p63 morpholino in zebrafish embryos resulted in mild (reduced fins) and severe (no fins) phenotypes. Accordingly, the expression of *GFP*, *zdlx5a* and *zdlx6a* is either reduced or absent in p63 morphant embryos. (D) SHFM1-BS1 was cloned in a reporter construct carrying the *LacZ* gene to generate transgenic mice. Specific expression of *LacZ* in the AER was shown at E9.5 and E15. Mutations in the p63 binding motif in SHFM1-BS1 disrupted the specific expression pattern in mice (E15). (E) A diagram shows chromosomal locations of SHFM1-BS1 and *Dlx5/Dlx6*. PCR primers used in the 3C experiment are indicated. (F) Three-dimensional physical interaction of SHFM1-BS1 to *Dlx5/Dlx6* was analysed by Chromosome Conformation Capture (3C) technique in mouse embryonic limb tissues (E10 and E15). SHFM1-BS1 interacts strongly to the *Dlx6* gene, as interaction frequency was clearly higher at the *Dlx6* gene than at surrounding regions. Interaction frequency is expected to attenuate over distance to SHFM1-BS1 if there is no active interaction. Error bars represent standard errors (N = 8 or 11 for E10 or E15 limbs, respectively).

To understand gene expression controlled by enhancer elements in embryonic limb development, we tested SHFM1-BS1, -BS2 and -BS3 in a transgenic reporter assay in zebrafish. A specific expression pattern of the *GFP* reporter controlled by SHFM1-BS1 but not by SHFM1-BS2 and -BS3 (data not shown) was observed in the AER and weakly in the ear and in the forebrain (Figure 5A). Expression of p63 detected by *in-situ* hybridization was only clearly localized to the AER (Figure 5B). The reporter expression promoted by the SHFM1-BS1 in the AER that directs growth and patterning of limbs and fins correlated perfectly with the expression of *p63, Dlx5 and Dlx6* during embryonic fin or limb development (Figure 5C) [14]. To further determine whether gene expression regulated by SHFM1-BS1 depends on p63 in zebrafish, we examined the enhancer activity of SHFM1-BS1 in *p63*-knockdown embryos injected with a specific p63 morpholino [4]. In p63-morphant embryos at 48 hours post fertilization (hpf), the fin buds were severely reduced (mild) or absent (severe) (Figure 5C), as reported previously [3], [4]. In the mild phenotypes, the expression of *GFP* induced by the enhancer was strongly reduced, as was the expression of the *zdlx5a* and *zdlx6a* genes. No fin defects were observed in embryos injected with a control morpholino (data not shown). Enhancer activity of SHFM1-BS1 was also tested in transgenic reporter assays in mice. Consistent with the zebrafish data, specific expression was observed in the AER in mouse embryos (E9.5 and E15), and the expression was lost when the p63-binding motif was mutated in SHFM1-BS1 (Figure 5D). These data showed that the specific expression in AER is dependent on p63. Taken together, our data obtained from animal models clearly demonstrated that SHFM1-BS1 can function as an enhancer element to control gene expression during embryogenesis and its activity is dependent on p63.

Having shown that gene expression regulated by SHFM1-BS1 correlates with that of *Dlx5* and *Dlx6* in zebrafish and mice, we tested whether SHFM1-BS1 physically interacts with the *Dlx5* and *Dlx6* promoters. To do that, we used the Chromosome Conformation Capture technique (3C) [56] that allows detecting the three-dimensional proximity of two chromosomal locations (Figure 5E and 5F and Figure S7). In mouse embryonic limb tissues (E10), the interaction frequencies of SHFM1-BS1 with the promoter of *Dlx6* and with the intergenic region between *Dlx5* and *Dlx6* were clearly higher than with the surrounding regions. This indicates that SHFM1-BS1 indeed strongly interacts with *Dlx6*. A weaker interaction of SHFM1-BS1 with *Dlx6* was also detectable in E15 limbs. In addition, SHFM1-BS1 appeared to interact with the intergenic region between *Dlx5* and *Dlx6* that contains highly conserved enhancer elements [57], [58]. We did not observe clear interaction of SHFM1-BS1 with the promoter of *Dlx5*. Taken together, our data show that p63 binding sites identified in HKCs can function as regulatory elements to control gene expression in embryonic limb development. We further conclude that disruption of regulation of *DLX5* and *DLX6* controlled by p63 likely causes SHFM1.

## Discussion

In this study, we established the DNA-binding profiles of p63 in a physiologically relevant human cellular system to identify target genes and regulatory elements controlled by p63. We show that one of the identified p63 binding sites acts as a cis-regulatory element to control gene expression in the AER that correlates perfectly with the expression pattern of *DLX6* and *DLX5* during embryonic development. A novel microdeletion that includes this binding site but leaves *DLX5* and *DLX6* intact leads to SHFM.

With a prevalence of 2–6% in humans, congenital malformations represent a major medical problem [59]. Elucidation of the genetic basis of this heterogeneous group of disorders is important for genetic counseling and for basic research. Although current main stream genetic studies still focus on mutations in the coding regions of genes, disease mechanisms associated with genetic variants in short- or long-range regulatory elements are increasingly recognized. Consistent with regulatory elements being required for correct spatio-temporal expression of developmental genes [60], mutations in non-coding cis-regulatory elements have been reported to cause congenital defects and have emerged as a disease mechanism [61]–[64]. Evolutionary conservation can be a powerful tool in the identification of regulatory elements [65]–[67]. A recent study identified a number of highly-conserved elements surrounding the *IRF6* gene which is known to be involved in several types of syndromic and non-syndromic cleft lip/palate [68]. In one of these elements a SNP that affects an AP-2alpha binding site was identified to associate with increased risk of cleft lip. This conserved element was able to drive the expression of a reporter gene during mouse orofacial development. Interestingly, in our ChIP-seq study we identified the very same cis-regulatory element as a strong p63 binding site that functions as an enhancer element to control expression of *IRF6* [69]. However, as only ultra-conserved elements are the focus of the evolutionary conservation approach, not all important regulatory elements can be identified. For example, conservation analysis in vertebrates of the enhancer element SHFM1-BS1 in our study was not found as an ultra-conserved element (Figure S8 and data not shown), even though it is well conserved. In addition, the identity of the transcription factors controlling regulatory elements may not always be derived from the genomic sequences. Our functional strategy of genome-wide p63 binding profiling does not depend on motif prediction or evolutionary conservation and reveals a large number of potential cis-regulatory elements controlled by p63.

We used human keratinocytes for our studies as recent work on transcription factor p53 revealed that responsive elements are not always conserved across species [70]. Moreover, primary HKC cell lines represent a cell type that is highly significant for p63-associated disorders. As many as 43% of the binding sites (2510 out of 5807) from a p63 binding dataset using the ChIP-on-chip technique in a cervical carcinoma cell line [17] were also present in our ChIP-seq dataset. Given that different cell types and techniques were used in these studies, the overlap of these two datasets is remarkable. Nevertheless, our data from HKCs are highly reliable (Figure S1, Table S1) and appear to represent functional p63 binding sites more accurately [17].

Similar to recent reports on DNA-binding profiles of other gene-specific transcription factors [71]–[74], the number of identified p63 binding sites is large, which was not predicted by the classical paradigm of gene transactivation. Our extensive bioinformatic analyses suggest that the majority of the identified p63 binding sites are biologically functional, as 94% of the binding sites contain a p63 consensus motif, and evolutionary conservation (Figure 2) and phenotypic similarity of PTG-associated diseases (Table 2) are significantly higher than random expectation. The binding sites are frequently located in intronic regions or at a distance from promoters. Thus gene-specific transcription factors may not only activate transcription at proximal promoters but also regulate gene expression at a distance perhaps by looping mechanisms. A recent report on the chromatin interaction map of the Oestrogen-Receptor-α (ERα) [75] also found long-range interaction of ERα binding sites and their target genes. This proposed looping mechanism is consistent with the notion that SHFM1-BS1 physically interacts with the *DLX5/DLX6* genes that are located more than 250kb downstream from SHFM1-BS1 (Figure 5E and 5F). Furthermore, binding sites identified in a certain cell type may also represent target genes and regulatory elements that can be regulated at different developmental stages in other cells and tissues. For example, SHFM1-BS1 was identified in human adult skin keratinocytes where *DLX5/DLX6* are moderately expressed and their expression is not altered in EEC patient keratinocytes (our unpublished data). Nevertheless, SHFM1-BS1 can drive gene expression in the AER during early embryonic limb development.

It has been well established that p63 plays an important role in limb development, as mutations in *p63* give rise to limb defects in complex syndromes as well as to isolated SHFM (SHFM4) [21]. In this report, our data strongly indicate that p63 plays a role in SHFM1 by regulating *DLX5/DLX6* through SHFM1-BS1 that physically interacts with the *Dlx6* promoter in the AER (Figure 5F). *DLX5* and *DLX6* were previously reported as target genes of p63 as p63 binds to *Dlx5/Dlx6* promoters and activates these genes [14]. However, we did not detect p63 binding sites at the promoter regions of these two genes in our HKCs (Figure 3). It is plausible that looping of SHFM1-BS1 to the promoters may result in a binding signal in a ChIP experiment. We also did not observe p63 activation on the *Dlx5/Dlx6* promoters in transient transfection assays (Figure 4E and data not shown). In addition, the SHFM4 mutations only affected transactivation mediated by SHFM1-BS1 moderately in our transfection assay using Saos2 cells which do not express any endogenous p53 and p63 (Figure 4C). The disruption of activation on *Dlx5/Dlx6* promoter was previously reported in transfection assays using U2OS cells where endogenous wild type p53 is expressed [14]. The use of different cells in transfection assays may be responsible for the variable results of transactivation assays. Moreover, our observations suggest that SHFM4 mutations that do not directly affect DNA-binding might disrupt protein-protein interaction or DNA looping to the *Dlx5/Dlx6* promoters to abolish transactivation. Importantly, we showed that the enhancer element SHFM1-BS1 activates gene expression in the AER during embryogenesis and that this activation is p63 dependent.

In addition to the functional data in model systems, we provide genetic data that support an important role for the enhancer element SHFM1-BS1 in limb development by the identification of a novel microdeletion 7q21 in an SHFM patient. This is a unique microdeletion, as the reported deletions in the SHFM1 patients so far all contain SHFM1-BS1 and *DLX5*/*DLX6* (Figure S5) [22], [28], [47]–[51], [76]–[81]. Within the novel deletion, *DYNC1I1 and SLC25A13* are unlikely to play a role in limb development [52], [53]. The other gene within the minimal deletion is *DSS1*. *DSS1* is expressed in the mesenchyme of the developing mouse limb [48], and the causative role of *DSS1* in SHFM1 has not been demonstrated. Moreover, the expression of *DSS1* in limb bud mesenchyme remains normal in *DLX5/DLX6^−/−^* mice displaying typical SHFM phenotypes [34]. Our functional analyses support the notion that the enhancer element SHFM1-BS1 regulates expression of *DLX6* and possibly *DLX5*, and that loss of this gene regulation gives rise to SHFM1. This model is in agreement with recent reports on genomic aberrations in 7q21 that were associated with SHFM1. In one report, a human breakpoint located at 38 kb telomeric to *DSS1* and at 258 kb centromeric to *DLX6* is associated with SHFM and hearing loss phenotype (Figure S5) [82]. This breakpoint leaves the SHFM1-BS1 association with *DSS1* intact, but disconnects it from *DLX5* and *DLX6*. Interestingly in this translocation, the chromosomal context between the p63 binding sites SHFM1-BS2 and -BS3 with *DLX5/DLX6* is not affected, which suggests that SHFM1-BS2 and -BS3 do not play a role in SHFM1. Another report identified a familial paracentric inversion-deletion 7q21 that affected a potential enhancer element (Figure S5) [83]. However, the spatio-temporal expression mediated by the identified element in this report did not support a role in limb development. Therefore, it is more likely that the SHFM phenotype in this family is due to the dissociation of the *DLX5/DLX6* genes from SHFM1-BS1 by the inversion. It should be noted that in the same report, a 5,115 bp deletion (chr7:96,402,577–96,407,691, hg18) was identified at the breakpoint. We did not observe a p63 binding site in this deletion (Figure 5S). Our results and those from others thus support the hypothesis that SHFM1-BS1 plays an essential role in the regulation of *DLX5/DLX6*. A genetic approach to delete SHFM1-BS1 in mice can give an unambiguous demonstration of its role to control expression of *Dlx5* and *Dlx6*. Intriguingly, whereas *Dlx5/Dlx6* are expressed in the craniofacial region at later stages of development (E14–17 in mice) [29], [34], absence of specific expression controlled by SHFM1-BS1 in craniofacial regions indicates that SHFM1-BS1 is not a regulatory element for orofacial development (data not shown). Different enhancer elements may regulate *DLX5* and *DLX6* in these tissues. It will be of interest to test other less conserved p63 binding sites within the CTCF boundaries for a role in craniofacial development.

In summary, we have identified binding sites of p63 and taken the first step to build a gene network regulated by p63 with ChIP-seq analysis in human primary keratinocytes. Our study provides potential target genes as well as high-resolution regulatory elements relevant to *p63*-related diseases. Reporter assays in a large scale to test p63 binding sites in the animal models will provide valuable information on functions of p63 target genes in ectodermal development. Our findings strongly indicate that loss of the regulatory element SHFM1-BS1 identified by a p63 binding site constitutes a novel disease mechanism responsible for SHFM1. Identified target genes and regulatory elements of p63 can therefore be analysed for mutations and microdeletions to understand the disease mechanisms of unresolved diseases that resemble p63-associated syndromes.

## Materials and Methods

### Ethics statement

All procedures regarding establishing human primary keratinocytes were approved by the ethical committee of the Radboud University Nijmegen Medical Centre (“Commissie Mensgebonden Onderzoek Arnhem-Nijmegen”). Informed consent was obtained. All animal work has been conducted according to relevant national and international guidelines.

### Clinical summary of the SHFM1 patient

The patient was born with bilateral foot anomalies and had no other dysmorphic features, in particular no hand anomalies, evidence of ectodermal dysplasia, scalp defects, oral cleft, bifid uvula, tear duct anomalies, eyelid adhesions or abnormal nails. On review at age 2 years and 7 months of age, she was healthy and was well grown and development was within normal limits.

### Human primary keratinocyte culture

Skin biopsies were taken from the trunk of healthy volunteers to set up the primary keratinocyte culture [84]. Keratinocyte cultures in Keratinocyte Growth Medium (KGM) under undifferentiated condition were previously described [85].

### ChIP and ChIP–seq

Human primary keratinocytes under proliferating condition where p63 is expressed at the highest level were used for ChIP and ChIP-seq analysis. Cells were crosslinked with 1% formaldehyde for 10 minutes and chromatin was collected as described [86]. Chromatin was sonicated using a Bioruptor sonicator (Diagenode) for 2 times of 8 minutes at high power, 30s ON, 30s OFF. p63 antibodies 4A4 (Abcam) and H129 (Santa Cruz) were used in ChIP-qPCR and ChIP-seq analyses. ChIP experiments were performed as previously described [37]. ChIP-seq analysis was performed on a Solexa Genome Analyzer (Illumina) as described previously [71].

### ChIP-seq data analysis

All 32-bp sequence reads were uniquely mapped to the human genome NCBI build 36.1 (hg18) with zero or one mismatch using ELAND (Illumina), resulting in 3.2, 6 and 20 million unique reads for the three analyzed samples, wt1 with 4A4 ChIP, wt2 with 4A4 ChIP and wt2 with H129 ChIP, respectively. Peak recognition was performed using MACS [35] with default settings and a *P* value threshold of 1E-9, giving 18,133, 14,963 and 29,166 peaks in ChIP-seq tracks of wt1 with 4A4 ChIP, wt2 with 4A4 ChIP and wt2 with H129 ChIP, respectively. Peaks were mapped to RefSeq genes, downloaded from the UCSC Genome Browser (hg18), to determine genomic location. The ChIP-seq data and associated peaks have been deposited in NCBI's Gene Expression Omnibus [87] and are accessible through GEO Series accession number GSE17611 (http://www.ncbi.nlm.nih.gov/geo/query/acc.cgi?acc=GSE17611).

### *De novo* motif search

To determine the p63 motif, a *de novo* motif prediction pipeline combining three motif prediction tools, MotifSampler [88], Weeder [89] and MDmodule [90], was run on 2273 (20%) randomly selected 200-bp peak sequences (centered at the peak summit as reported by MACS) and PWMs were generated. We used the ‘large’ analysis setting for Weeder. MDmodule and MotifSampler were each used to predict 10 motifs for each of the widths between 6 and 20. The significance of the predicted motifs was determined by scanning the remaining 80% of the peak sequences and two different backgrounds: a set of random genomic sequences with a similar genomic distribution as the peak sequences and a set of random sequences generated according to a 1st order Markov model, matching the dinucleotide frequency of the peak sequences. *P* values were calculated using the hypergeometric distribution with the Benjamin-Hochberg multiple testing correction. All motifs with a *P* value<0.001 and an absolute enrichment of at least >1.5-fold compared to both backgrounds were determined as significant. We calculated the ROC AUC for all significant motifs and chose the best performing motif based on the ROC AUC (See Table S2 for the results). The PWM of this motif was combined with the p53scan algorithm to generate p63scan, using an optimal threshold, determined by the maximum f-measure as described previously [37]. The p63scan algorithm can be downloaded from http://www.ncmls.eu/bioinfo/p63scan/. To examine the correlation of motif score and peak height, all peaks were divided in quartiles according to peak height (the number of reads per peak). For each quartile the distribution of the motifs score as determined by p63scan is depicted as a boxplot.

### Motif over-representation analysis using TRANSFAC

To detect putative transcription factor motifs reported in the TRANSFAC Professional database version 2009.3 [39], the MotifScanner program [91] was used. The search was performed on both strands using a 3rd-order Markov model calculated from the human promoter set of the Eukaryotic Promoter Database (EPD) as a background model. The parameter *p* (a prior probability of finding one instance of the motif in a sequence) was set to a value of 0.5. To identify motifs that are overrepresented in the p63 motif-less binding sites, the binomial test was used. The obtained *P* values were corrected for multiple testing (631 motifs for which sites were found in the p63-binding regions) using a Bonferroni correction.

### Quantitative PCR

Quantitative PCR primers were designed using Primer3 (http://frodo.wi.mit.edu) [92], and qPCR reactions were performed in the 7500 Fast Real Time PCR System apparatus (Applied Biosystems) by using iQ SYBR Green Supermix (Biorad) according to the manufacturer's protocol. For qPCR of ChIP analysis, one primer set was used for each tested binding region (Table S8) and ChIP efficiency of certain binding sites was calculated using percentage of ChIPped DNA against input chromatin.

### Analysis of potential target genes associated with phenotypic defects using human and mouse disease bases

Text mining-based [43] feature overrepresentation and gene to disease mapping were determined using the Online Mendelian Inheritance in Man (OMIM) disease database [93], [94]. Detailed information can be found in supplementary information. Human diseases associated with SHFM were taken from the Pictures Of Standard Syndromes and Undiagnosed Malformations (POSSUM) database [44], current as of August 2007, and mapped to genes through their OMIM IDs. Mouse SHFM-associated phenotypes and associated genes were taken from the Jackson Laboratory's Mouse Genome Database (http://www.informatics.jax.org/) [45].

### Evolutionary conservation of binding sites

To assess the evolutionary conservation of the 11,369 sites bound by p63, the PhastCons [54] conservation track from the UCSC Genome Browser was used to calculate PhastCons Conservation Score (PCCS). Conservation based on 44 vertebrate genomes was chosen because the p63 gene has 1-1 orthologs throughout the vertebrates [42]. The conservation for a region was calculated as the average conservation of each nucleotide therein. To analyse the correlation of PCCS and peak height, all peaks were divided in quartiles according to peak height (the number of reads per peak). For each quartile the distribution of the PhastCons Conservation Scores (PCCS) is depicted as a boxplot.

### Mapping deletion in a SHFM patient using ultra-high comparative genomic hybridization

For detailed detection of chromosome 7 aberration, high resolution NimbleGen HG18 chromosome 7 specific 385K arrays were used (B3738001-00-01; Roche NimbleGen Systems, Madison, Wisconsin, USA). The 385K average probe distance was 365bp. DNA labeling, array hybridization, post-hybridization washes and scanning were performed according to the manufacturer's instructions (Roche NimbleGen). The acquired images were analyzed using NimbleScan V2.4 extraction software (Roche NimbleGen). For each spot on the array, the log2 Cy3/Cy5 ratio (relative intensity of the Cy3 labeled patient DNA vs. the Cy5 labeled male DNA reference pool of 5 healthy male individuals) was calculated using the segMNT algorithm, which also applied an automatic segment detection. A 50× averaging window was generated, resulting in 20kb segments for this array. Breakpoints were determined with SignalMap V1.9 software (Roche NimbleGen) and 20kb averaged log2 ratios were visualized in the UCSC genome browser.

### Constructs and transactivation assays

The genomic regions of p63 binding site peaks were amplified by PCR with gateway cloning primers and cloned into a modified *ccdB*-containing pGL3-Enhancer Vector, or a *ccdB*-containing pGL3-Promoter Vector, or a *ccdB*-containing pGL3-*Dlx5* Vector. The *ccdB*-containing pGL3-*Dlx5* Vector was generated by amplification of mouse genomic DNA using primers described in Table S8 to obtain the mouse *Dlx5* promoter to replace the SV40 promoter with BglII and HindIII sites in the *ccdB*-containing pGL3-Promoter Vector. Point mutations were introduced into p63-binding motifs of SHFM1-BS1 to generate mutant p63 binding sites, where the essential cytosine and guanine bases were mutated to adenosine. The ΔNp63α wild-type (pcDNA-mM_ΔNp63α) expression plasmid has been described previously [85]. Point mutations were introduced into this plasmid to generate R204W, R279H, R304W, K194E and L517F mutations. Transfection and luciferase assays were described previously [85]. All cloning and mutagenesis primers are described in Table S8.

### Transgenic reporter analyses in zebrafish and mice

Human genomic fragments containing the SHFM1-BS1, -BS2 and -BS3 were amplified with primers described in Table S8. The PCR fragments were subcloned in PCR8/GW/TOPO vector and then transferred, through recombination using Gateway technology, to the ZED destination vector for zebrafish transgenesis [95]. This vector contains the *Xenopus* Cardiac actin promoter driving DsRed as a positive control for transgenesis. To generate the zebrafish transgenic embryos, we used Tol2 transposon/transposase method [96] with minor modifications. Volume of 2–5nl of mixture containing 25ng/ul of transposase mRNA, 20ng/ul of phenol/chloroform purified ZED constructs and 0.05% phenol red was injected in the cell of 1 cell stage embryos. Three or more independent stable transgenic lines were generated for each construct. For the generation of transgenic mice, the genomic fragments with and without point mutations in p63 consensus motif were transferred into a vector containing the human minimal beta-globin promoter, *lacZ* and a SV40 polyadenylation signal. Constructs were linearized and the vector backbone removed prior to microinjection into the pronucleus of one-cell mouse embryos. F0 embryos of 9.5–13 dpc stages were harvested and stained for *lacZ* activity.

### Morpholino injections and in situ hybridizations in zebrafish embryos

Once cell stage embryos were injected with 3ng of ΔNp63 MO II (TCCACAGGCTCCAGGATTCTTACCC) as described previously [4]. Injected embryos were raised at 28°C in standard E3 medium and fixed at 48 hours post fertilization in 4% paraformaldehyde overnight at 4°C. In situ hybridizations were carried out as described [97]. As a control, we injected a similar amount of a MO directed against the *Xenopus tropicalis olig2* gene that shows no match in the zebrafish genome [98].

### Chromosome Conformation Capture (3C) assay

Chromosome Conformation Capture (3C) assay was performed as referred in Hagege et al., 2007 [56]. Limbs of E10- and E15-stage mouse embryos were dissected and processed to get single cells preparations. Ten million isolated cells were first fixated with 2% formaldehyde, and then cells were lysed and nuclei were digested with HindIII endonuclease (Roche). After that, DNA was ligated with T4 DNA ligase (Promega) in low concentration conditions to favour intramolecular ligations. A set of locus specific primers close to a HindIII site (Table S8) was designed with Primer3 *v. 0.4.*0 [92]. These primers were used to make semi-quantitative PCRs to measure the relative enrichment in each ligation product. The primer near to the BS1 enhancer was taken as the fixed primer, and the different interactions were tested using primers close to the promoters of *DLX5* and *DLX6* genes. For each interaction two negative control primers were designed about 30 kb upstream and downstream the promoter specific primer. PCR products were run in agarose gels and measured using a Typhoon scanner. Product values were related to a control composed of two BACs that encompass our region of interest.

## Supporting Information

Figure S1 ChIP-qPCR analysis of p63 binding in human primary keratinocytes using two different p63 antibodies 4A4 (pan-p63) and H129 (α-specific). Specific binding of p63 to the tested binding sites was observed, including to binding sites at p21^WAF/CIP19^ and DST which served as positive controls, but not to the negative controls myoglobin exon 2 (myo) and a no-gene region (chr11).
(0.16 MB PDF)

Figure S2 Correlation of motif scores to peak heights. All peaks were divided in quartiles according to peak height (the number of reads per peak). For each quartile the distribution of the motifs score as determined by p63scan is depicted as a boxplot.
(0.16 MB PDF)

Figure S3 Genomic distribution of p63 binding sites with and without p63 consensus binding motifs.
(0.13 MB PDF)

Figure S4 Correlation of PhastCons Conservation Scores (PCCS) to peak heights and p63 binding motifs of the p63 binding sites. (A) The percentage of p63 binding sites (y-axis) is plotted against decreasing cut-off values of the PhastCons Conservation Score (PCCS) (x-axis) for two groups of peaks: those with a p63 motif and those without a p63 motif, as determined by p63scan. (B) All peaks were divided in quartiles according to peak height (the number of reads per peak). For each quartile the distribution of the PhastCons Conservation Scores (PCCS) is depicted as a boxplot.
(0.08 MB PDF)

Figure S5 Previously reported chr.7 deletions involved in SHFM1 (hg18). (A) The grey track CGH array CNV data with deletion in the SHFM1 patient is compared to the minimum critical regions for SHFM1 based on the literature. Pale green tracks represent deleted intervals in patients with SHFM1 who have cytogenetic deletions (Del Porto *et al.*, 1983; Tajara *et al.*, 1989; Morey *et al.*, 1990; Roberts *et al.*, 1991; Nunes *et al.*, 1994; McElveen *et al.*, 1995; Montgomery *et al.*, 2000). Dark green tracks represent deleted intervals in patients with SHFM1 where mapping has been done with STS markers (Marinoni *et al.*, 1995; Crackower *et al.*, 1996; Fukushima *et al.*, 2003; Wieland *et al.*, 2004). Purple tracks represent summed mapping of deletions combined from many patients with SHFM1 (Scherer *et al.*, 1994; Tackels-Horne *et al.*, 2001). The brown track represent a microdeletion at the break point of a chromosome inversion in a patient with SHFM1 (Brown *et al.*, 2010). The red bar represents three p63 binding sites. (B) A zoomed-in view of the region including SHFM1-BS1, -BS2 and -BS3 and *DLX5/6*. A translocation in SHFM1 that disconnects SHFM1-BS1 with *DLX5/6* is depicted with a black arrow (Saitu *et al.*, 2009).
(0.24 MB PDF)

Figure S6 Genomic qPCR analysis of deletion of the p63 binding site SHFM1-BS1 in the SHFM1 patient. Genomic qPCR was performed to confirm the deletion in the SHFM1 patient revealed by CGH analysis. Copy number was calculated against an internal control primer set in the *CFTR* gene.
(0.18 MB PDF)

Figure S7 Raw data of 3C experiments in mouse limbs at E10 and E15.
(0.47 MB PDF)

Figure S8 Evolutionary conservation of the p63 binding site SHFM1-BS1 in vertebrates. SHFM1-BS1 was examined for its conservation in vertebrates using USCS genome browser. The consensus motif of p63 is highlighted in the red box.
(0.19 MB PDF)

Table S1 Validation by ChIP-qPCR of detected binding sites in ChIP-seq analysis.
(0.07 MB DOC)

Table S2 Motif analysis with a *de novo* motif prediction pipeline.
(0.02 MB XLS)

Table S3 The motifs significantly overrepresented in the p63 motif-less binding sites.
(0.03 MB DOC)

Table S4 The most significant GO annotation terms of potential target genes of p63.
(0.05 MB DOC)

Table S5 OMIM IDs associated with genes containing p63 binding sites.
(0.14 MB XLS)

Table S6 Affected features in p63 potential target gene-associated diseases (PTG-associated diseases).
(0.37 MB XLS)

Table S7 Genes potentially involved in SHFM mouse models or human SHFM phenotypes.
(0.04 MB DOC)

Table S8 Primers for expression, ChIP analyses, and for cloning.
(0.03 MB XLS)
