# Supplementary material for: Genome-Wide Profiling of p63 DNA–Binding Sites Identifies an Element that Regulates Gene Expression during Limb Development in the 7q21 SHFM1 Locus
Source: PLoS Genet. 2010 Aug 19;6(8):e1001065. doi: 10.1371/journal.pgen.1001065 (PMC2924305; doi:10.1371/journal.pgen.1001065)
Supplement: Figure S1 — ChIP-qPCR analysis of p63 binding in human primary keratinocytes using two different p63 antibodies 4A4 (pan-p63) and H129 (α-specific). Specific binding of p63 to the tested binding sites was observed, including to binding sites at p21WAF/CIP19 and DST which served as positive controls, but not to the negative controls myoglobin exon 2 (myo) and a no-gene region (chr11). (0.16 MB PDF) [file pgen.1001065.s001.pdf]

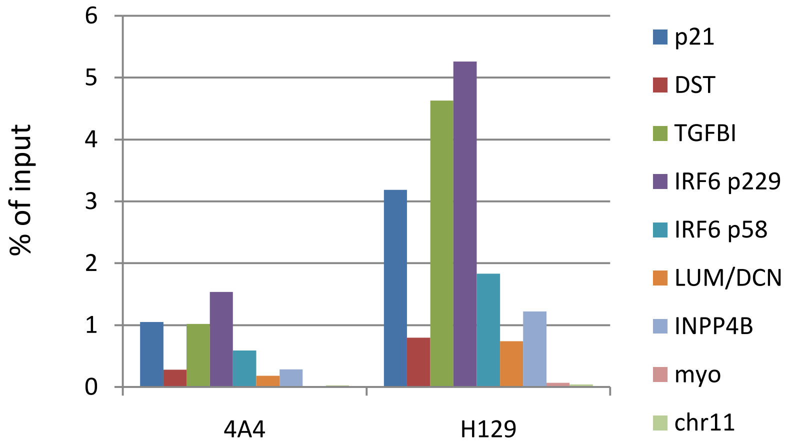

Figure S1. ChIP-qPCR analysis of p63 binding in human primary keratinocytes using two different p63 antibodies 4A4 (pan-p63) and H129 (α-specific). Specific binding of p63 to the tested binding sites was observed, including to binding sites at p21$^{WAF/CIP19}$ and DST which served as positive controls, but not to the negative controls myoglobin exon 2 (myo) and a no-gene region (chr11).
