# Supplementary material for: Genome-Wide Profiling of p63 DNA–Binding Sites Identifies an Element that Regulates Gene Expression during Limb Development in the 7q21 SHFM1 Locus
Source: PLoS Genet. 2010 Aug 19;6(8):e1001065. doi: 10.1371/journal.pgen.1001065 (PMC2924305; doi:10.1371/journal.pgen.1001065)
Supplement: Figure S2 — Correlation of motif scores to peak heights. All peaks were divided in quartiles according to peak height (the number of reads per peak). For each quartile the distribution of the motifs score as determined by p63scan is depicted as a boxplot. (0.16 MB PDF) [file pgen.1001065.s002.pdf]

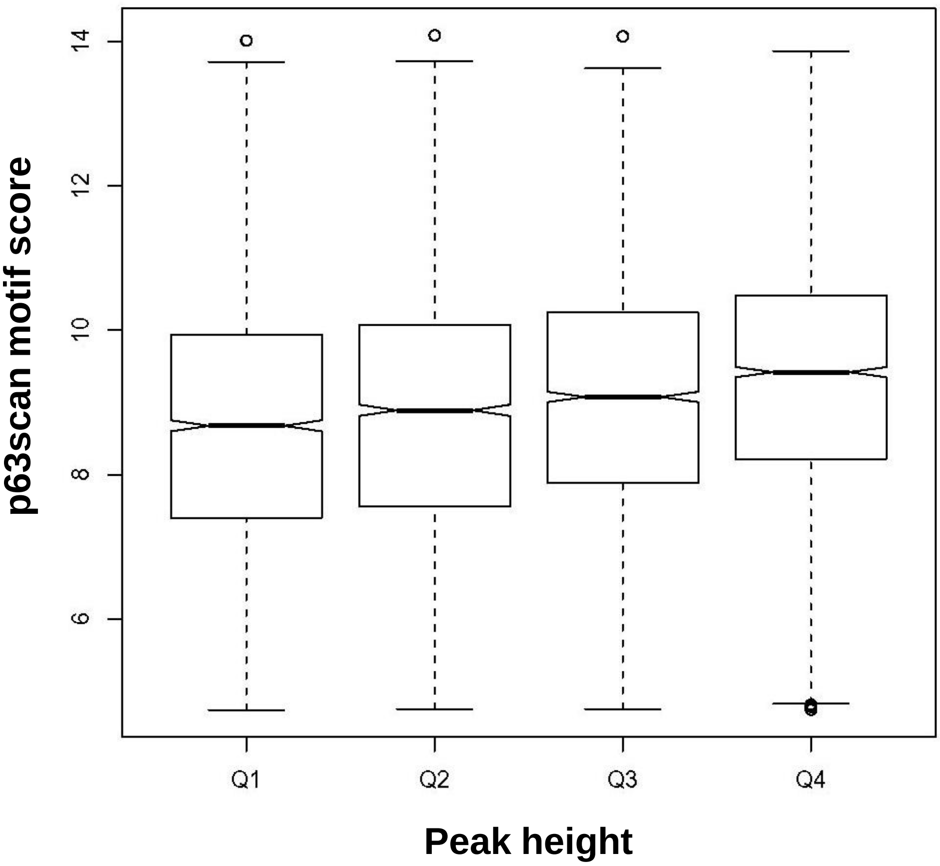

Figure S2. Correlation of motif scores to peak heights. All peaks were divided in quartiles according to peak height (the number of reads per peak). For each quartile the distribution of the motifs score as determined by p63scan is depicted as a boxplot.
