# Supplementary figures and images for: Genome-Wide Profiling of p63 DNA–Binding Sites Identifies an Element that Regulates Gene Expression during Limb Development in the 7q21 SHFM1 Locus
Source: PLoS Genet. 2010 Aug 19;6(8):e1001065. doi: 10.1371/journal.pgen.1001065 (PMC2924305; doi:10.1371/journal.pgen.1001065)

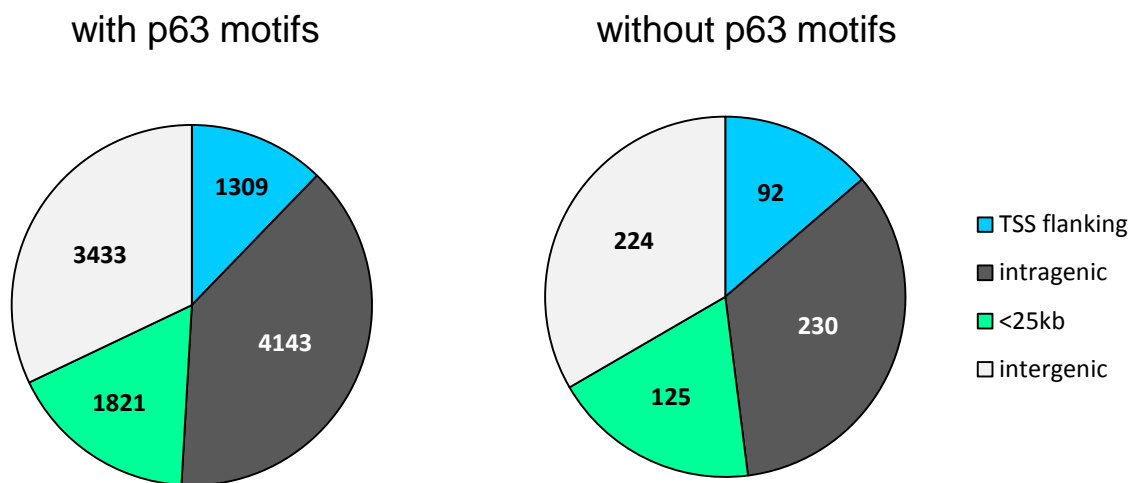

**Figure S3. Genomic distribution of p63 binding sites with and without p63 consensus binding motifs.**

Supplement: Figure S3 — Genomic distribution of p63 binding sites with and without p63 consensus binding motifs. (0.13 MB PDF) [file pgen.1001065.s003.pdf]

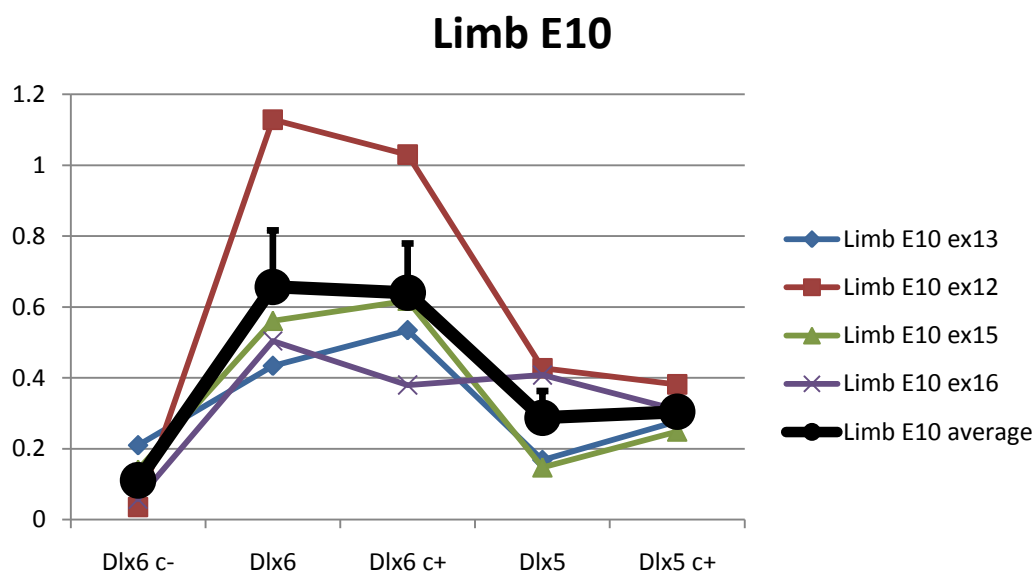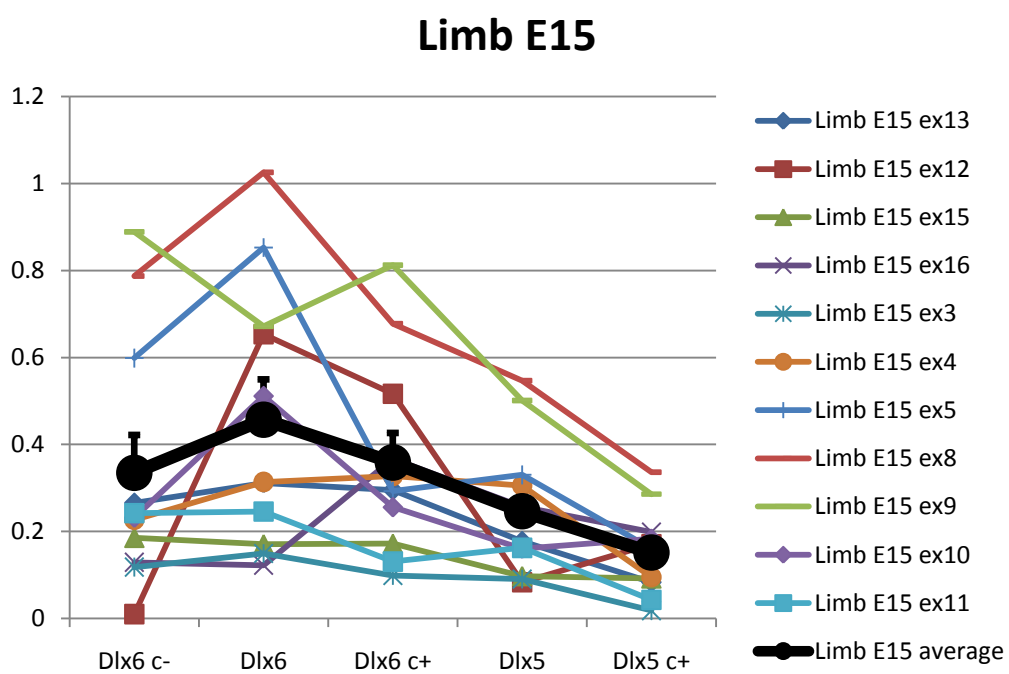

**Figure S7. Raw data of 3C experiments in mouse limbs at E10 and E15.**

Supplement: Figure S7 — Raw data of 3C experiments in mouse limbs at E10 and E15. (0.47 MB PDF) [file pgen.1001065.s007.pdf]
