# Supplementary material for: Genome-Wide Profiling of p63 DNA–Binding Sites Identifies an Element that Regulates Gene Expression during Limb Development in the 7q21 SHFM1 Locus
Source: PLoS Genet. 2010 Aug 19;6(8):e1001065. doi: 10.1371/journal.pgen.1001065 (PMC2924305; doi:10.1371/journal.pgen.1001065)
Supplement: Figure S4 — Correlation of PhastCons Conservation Scores (PCCS) to peak heights and p63 binding motifs of the p63 binding sites. (A) The percentage of p63 binding sites (y-axis) is plotted against decreasing cut-off values of the PhastCons Conservation Score (PCCS) (x-axis) for two groups of peaks: those with a p63 motif and those without a p63 motif, as determined by p63scan. (B) All peaks were divided in quartiles according to peak height (the number of reads per peak). For each quartile the distribution of the PhastCons Conservation Scores (PCCS) is depicted as a boxplot. (0.08 MB PDF) [file pgen.1001065.s004.pdf]

A

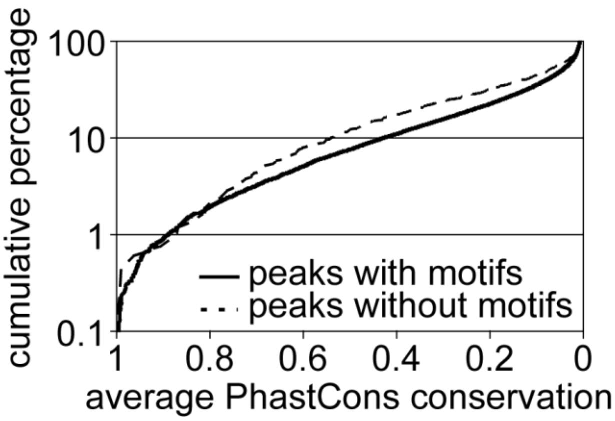

B

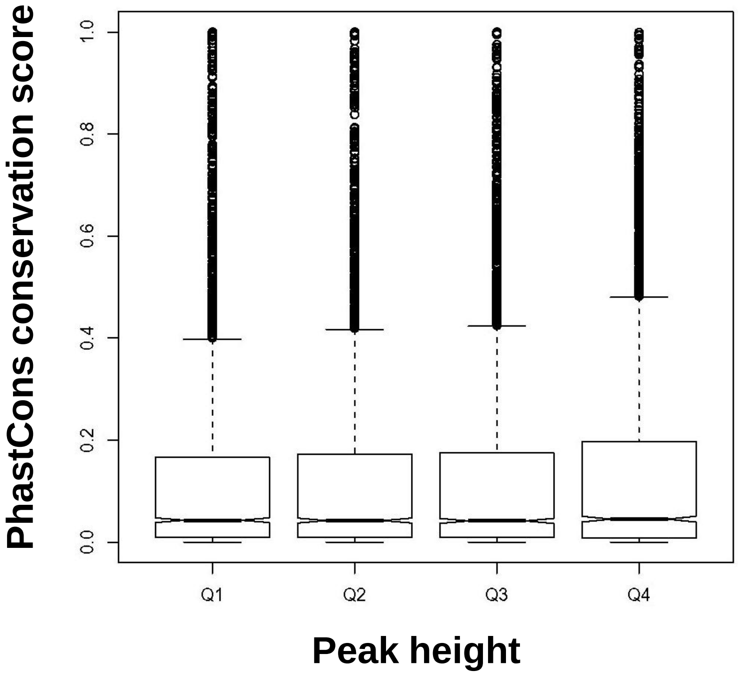

Figure S4. Correlation of PhastCons Conservation Scores (PCCS) to peak heights and p63 binding motifs of the p63 binding sites. A) The percentage of p63 binding sites (y-axis) is plotted against decreasing cut-off values of the PhastCons Conservation Score (PCCS) (x-axis) for two groups of peaks: those with a p63 motif and those without a p63 motif, as determined by p63scan. B) All peaks were divided in quartiles according to peak height (the number of reads per peak). For each quartile the distribution of the PhastCons Conservation Scores (PCCS) is depicted as a boxplot.
