# Supplementary material for: Genome-Wide Profiling of p63 DNA–Binding Sites Identifies an Element that Regulates Gene Expression during Limb Development in the 7q21 SHFM1 Locus
Source: PLoS Genet. 2010 Aug 19;6(8):e1001065. doi: 10.1371/journal.pgen.1001065 (PMC2924305; doi:10.1371/journal.pgen.1001065)
Supplement: Figure S5 — Previously reported chr.7 deletions involved in SHFM1 (hg18). (A) The grey track CGH array CNV data with deletion in the SHFM1 patient is compared to the minimum critical regions for SHFM1 based on the literature. Pale green tracks represent deleted intervals in patients with SHFM1 who have cytogenetic deletions (Del Porto et al., 1983; Tajara et al., 1989; Morey et al., 1990; Roberts et al., 1991; Nunes et al., 1994; McElveen et al., 1995; Montgomery et al., 2000). Dark green tracks represent deleted intervals in patients with SHFM1 where mapping has been done with STS markers (Marinoni et al., 1995; Crackower et al., 1996; Fukushima et al., 2003; Wieland et al., 2004). Purple tracks represent summed mapping of deletions combined from many patients with SHFM1 (Scherer et al., 1994; Tackels-Horne et al., 2001). The brown track represent a microdeletion at the break point of a chromosome inversion in a patient with SHFM1 (Brown et al., 2010). The red bar represents three p63 binding sites. (B) A zoomed-in view of the region including SHFM1-BS1, -BS2 and -BS3 and DLX5/6. A translocation in SHFM1 that disconnects SHFM1-BS1 with DLX5/6 is depicted with a black arrow (Saitu et al., 2009). (0.24 MB PDF) [file pgen.1001065.s005.pdf]

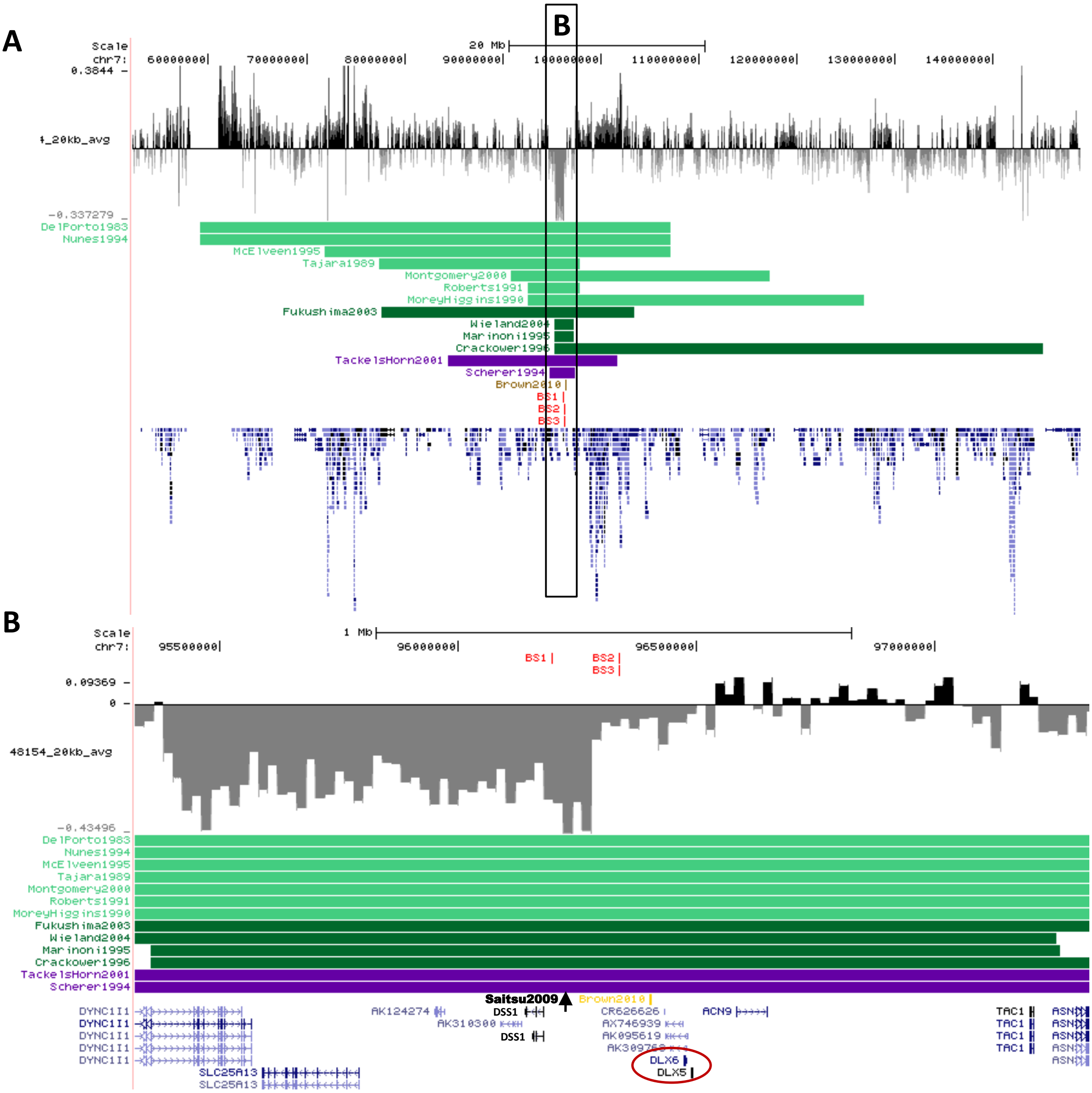

Figure S5. Previously reported chr.7 deletions involved in SHFM1 (hg18). A) The grey track CGH array CNV data with deletion in the SHFM1 patient is compared to the minimum critical regions for SHFM1 based on the literature. Pale green tracks represent deleted intervals in patients with SHFM1 who have cytogenetic deletions (Del Porto *et al.*, 1983; Tajara *et al.*, 1989; Morey *et al.*, 1990; Roberts *et al.*, 1991; Nunes *et al.*, 1994; McElveen *et al.*, 1995; Montgomery *et al.*, 2000). Dark green tracks represent deleted intervals in patients with SHFM1 where mapping has been done with STS markers (Marinoni *et al.*, 1995; Crackower *et al.*, 1996; Fukushima *et al.*, 2003; Wieland *et al.*, 2004). Purple tracks represent summed mapping of deletions combined from many patients with SHFM1 (Scherer *et al.*, 1994; Tackels-Horne *et al.*, 2001). The brown track represent a microdeletion at the break point of a chromosome inversion in a patient with SHFM1 (Brown *et al.*, 2010). The red bar represents three p63 binding sites. B) A zoomed-in view of the region including SHFM1-BS1, -BS2 and -BS3 and *DLX5/6.* A translocation in SHFM1 that disconnects SHFM1-BS1 with *DLX5/6* is depicted with a black arrow (Saitu *et al.*, 2009).
