# Supplementary material for: Genome-Wide Profiling of p63 DNA–Binding Sites Identifies an Element that Regulates Gene Expression during Limb Development in the 7q21 SHFM1 Locus
Source: PLoS Genet. 2010 Aug 19;6(8):e1001065. doi: 10.1371/journal.pgen.1001065 (PMC2924305; doi:10.1371/journal.pgen.1001065)
Supplement: Figure S6 — Genomic qPCR analysis of deletion of the p63 binding site SHFM1-BS1 in the SHFM1 patient. Genomic qPCR was performed to confirm the deletion in the SHFM1 patient revealed by CGH analysis. Copy number was calculated against an internal control primer set in the CFTR gene. (0.18 MB PDF) [file pgen.1001065.s006.pdf]

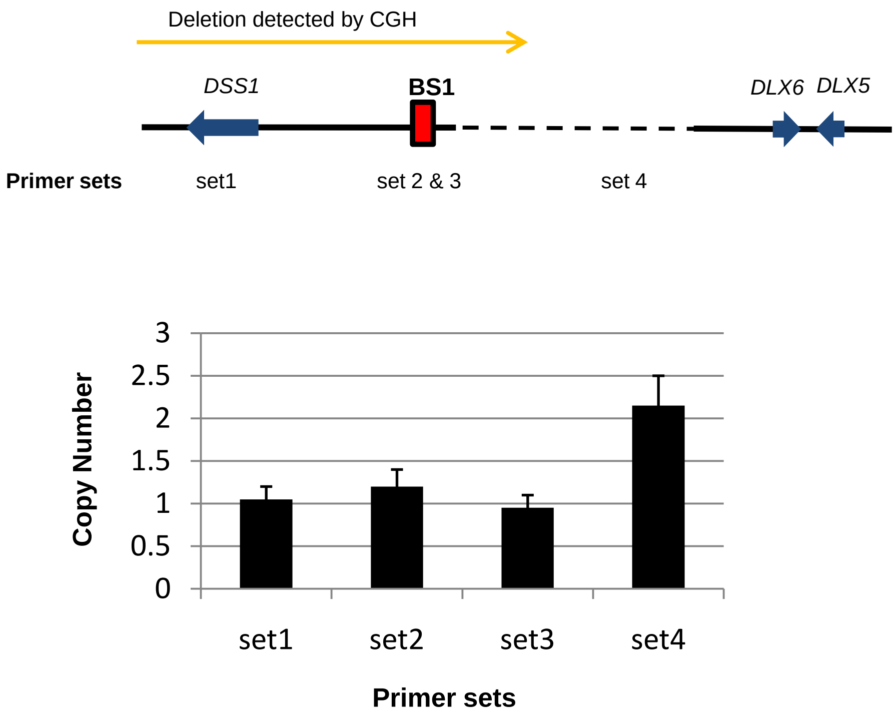

Figure S6. Genomic qPCR analysis of deletion of the p63 binding site SHFM1-BS1 in the SHFM1 patient. Genomic qPCR was performed to confirm the deletion in the SHFM1 patient revealed by CGH analysis. Copy number was calculated against an internal control primer set in the *CFTR* gene.
