# Supplementary material for: Genome-Wide Profiling of p63 DNA–Binding Sites Identifies an Element that Regulates Gene Expression during Limb Development in the 7q21 SHFM1 Locus
Source: PLoS Genet. 2010 Aug 19;6(8):e1001065. doi: 10.1371/journal.pgen.1001065 (PMC2924305; doi:10.1371/journal.pgen.1001065)
Supplement: Figure S8 — Evolutionary conservation of the p63 binding site SHFM1-BS1 in vertebrates. SHFM1-BS1 was examined for its conservation in vertebrates using USCS genome browser. The consensus motif of p63 is highlighted in the red box. (0.19 MB PDF) [file pgen.1001065.s008.pdf]

The conserved region in SHFM1-BS1, 96195551 – 96195750 (hg 18)

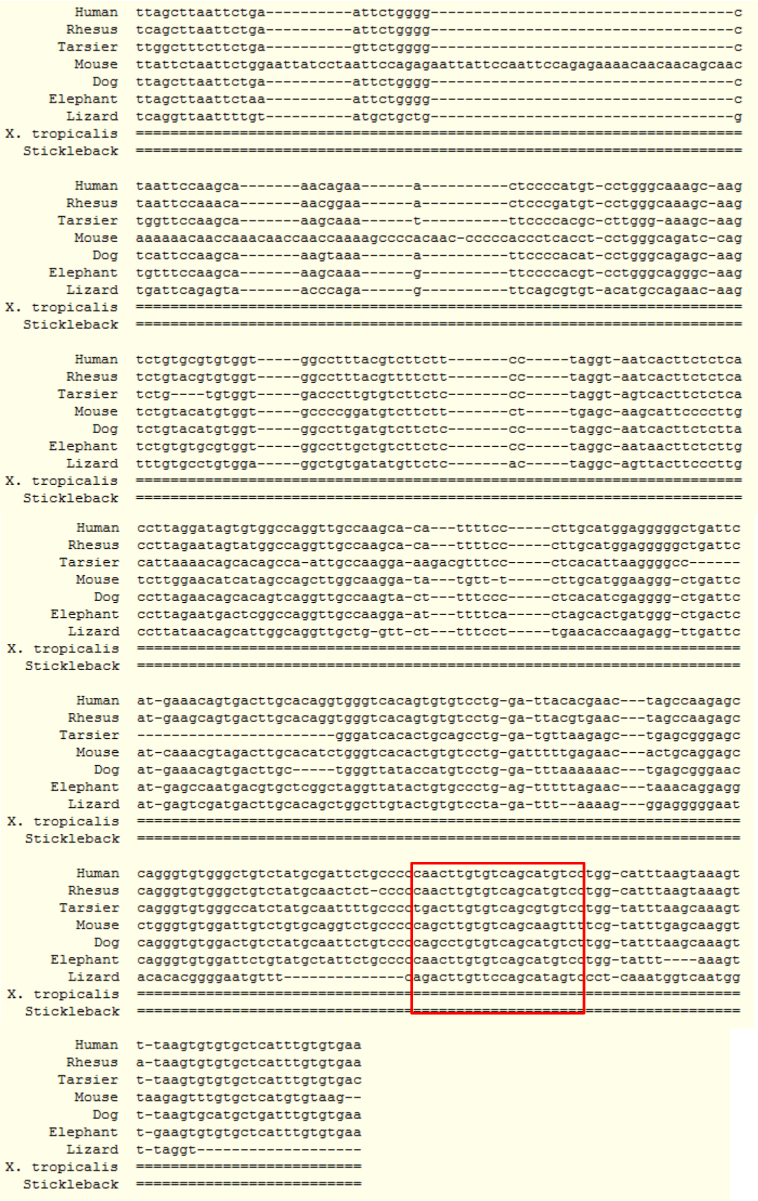

Figure S8. Evolutionary conservation of the p63 binding site SHFM1-BS1 in vertebrates. SHFM1-BS1 was examined for its conservation in vertebrates using USCS genome browser. The consensus motif of p63 is highlighted in the red box.
