# Supplementary material for: Genome-Wide Profiling of p63 DNA–Binding Sites Identifies an Element that Regulates Gene Expression during Limb Development in the 7q21 SHFM1 Locus
Source: PLoS Genet. 2010 Aug 19;6(8):e1001065. doi: 10.1371/journal.pgen.1001065 (PMC2924305; doi:10.1371/journal.pgen.1001065)
Supplement: Table S1 — Validation by ChIP-qPCR of detected binding sites in ChIP-seq analysis. (0.07 MB DOC) [file pgen.1001065.s009.doc]

**Table S1. Validation by ChIP-qPCR of detected binding sites in ChIP-seq analysis**

*Independent ChIP-qPCR experiments were performed to validate detected binding sites. Whether binding detected (+) or not (-) is based on the fold enrichment of 3 over an internal negative control regions to which p63 does not bind, myoglobin exon 2 region or a no-gene region on chromosome 11.
