# Supplementary material for: Genome-Wide Profiling of p63 DNA–Binding Sites Identifies an Element that Regulates Gene Expression during Limb Development in the 7q21 SHFM1 Locus
Source: PLoS Genet. 2010 Aug 19;6(8):e1001065. doi: 10.1371/journal.pgen.1001065 (PMC2924305; doi:10.1371/journal.pgen.1001065)
Supplement: Table S3 — The motifs significantly overrepresented in the p63 motif-less binding sites. (0.03 MB DOC) [file pgen.1001065.s011.doc]

Table S3: The motifs significantly overrepresented in the p63 motif-less binding sites

| **Transcription factor** | **Motif no. in the TRANSFAC database** | **p63 motif-containing sites** | **p63 motif-less binding sites** | **Corrected two-sided p-value** |
| --- | --- | --- | --- | --- |
| AP1* | 199 | 33.99 % | 53.97 % | 4∙10^-23^ |
| BACH1 | 495 | 9.28 % | 22.04 % | 7∙10^-20^ |
| BACH2 | 490 | 31.16 % | 48.88 % | 2∙10^-18^ |

***** For AP1, only the most significantly over-represented motif is reported. Nine other AP1 motifs were also significantly over-represented, with p-values (two-sided and corrected for multiple testing) ranging from 3∙10^-10^ to 6∙10^-16^.
