# Supplementary material for: Genome-Wide Profiling of p63 DNA–Binding Sites Identifies an Element that Regulates Gene Expression during Limb Development in the 7q21 SHFM1 Locus
Source: PLoS Genet. 2010 Aug 19;6(8):e1001065. doi: 10.1371/journal.pgen.1001065 (PMC2924305; doi:10.1371/journal.pgen.1001065)
Supplement: Table S4 — The most significant GO annotation terms of potential target genes of p63. (0.05 MB DOC) [file pgen.1001065.s012.doc]

**Table S4. The most significant GO annotation terms of** potential target genes of p63.

|  | **GO No.** | **Term** | **No. of genes** | ***P* value*** |
| --- | --- | --- | --- | --- |
| All genes  (10895) | GO:0009987 | cellular process | 4143 | 7.60E-24 |
|  | GO:0048856 | anatomical structure development | 1092 | 9.21E-13 |
|  | GO:0032502 | developmental process | 1329 | 4.74E-12 |
|  | GO:0048519 | negative regulation of biological process | 803 | 5.39E-12 |
|  | GO:0007155 | cell adhesion | 346 | 6.21E-12 |
|  | GO:0022610 | biological adhesion | 346 | 7.81E-12 |
|  | GO:0048731 | system development | 1000 | 8.47E-11 |
|  | GO:0016043 | cellular component organization | 1064 | 1.49E-10 |
|  | GO:0007275 | multicellular organismal development | 1206 | 1.95E-10 |
|  | GO:0010646 | regulation of cell communication | 480 | 2.05E-10 |
|  | GO:0007242 | intracellular signaling cascade | 569 | 2.10E-10 |
|  | GO:0048869 | cellular developmental process | 747 | 5.97E-10 |
| Genes with motifs  (10438) | GO:0009987 | cellular process | 3990 | 5.71E-25 |
|  | GO:0048856 | anatomical structure development | 1056 | 3.64E-13 |
|  | GO:0032502 | developmental process | 1287 | 8.03E-13 |
|  | GO:0007155 | cell adhesion | 336 | 3.73E-12 |
|  | GO:0022610 | biological adhesion | 336 | 4.68E-12 |
|  | GO:0048519 | negative regulation of biological process | 774 | 8.13E-12 |
|  | GO:0007242 | intracellular signaling cascade | 554 | 3.73E-11 |
|  | GO:0048731 | system development | 965 | 6.81E-11 |
|  | GO:0007275 | multicellular organismal development | 1166 | 6.86E-11 |
|  | GO:0048869 | cellular developmental process | 723 | 2.81E-10 |
|  | GO:0010646 | regulation of cell communication | 463 | 2.95E-10 |
| Genes without motifs  (944) | GO:0048872 | homeostasis of number of cells | 11 | 0.001205 |
|  | GO:0051173 | positive regulation of nitrogen compound metabolic process | 35 | 0.002269 |
|  | GO:0050793 | regulation of developmental process | 36 | 0.002614 |
|  | GO:0051094 | positive regulation of developmental process | 19 | 0.003227 |
|  | GO:0009891 | positive regulation of biosynthetic process | 36 | 0.004333 |
|  | GO:0007155 | cell adhesion | 36 | 0.004702 |

* *P* value was obtained using default setting in DAVID Bioinformatic Resources 6.7 (NIAID, NIH) without multiple testing.
