# Supplementary material for: Genome-Wide Profiling of p63 DNA–Binding Sites Identifies an Element that Regulates Gene Expression during Limb Development in the 7q21 SHFM1 Locus
Source: PLoS Genet. 2010 Aug 19;6(8):e1001065. doi: 10.1371/journal.pgen.1001065 (PMC2924305; doi:10.1371/journal.pgen.1001065)
Supplement: Table S7 — Genes potentially involved in SHFM mouse models or human SHFM phenotypes. (0.04 MB DOC) [file pgen.1001065.s015.doc]

**Table S7.** Genes potentially involved in SHFM mouse models or human SHFM phenotypes

| **Gene** | **Mouse model** | **Human SHFM (POSSUM)** |
| --- | --- | --- |
| BRCA2 | 0 | 1 |
| CDH3 | 0 | 1 |
| DLX5 | 1 | 0 |
| DLX6 | 1 | 0 |
| FBXW4 | 1 | 1 |
| FOXP2 | 0 | 1 |
| GJA1 | 0 | 1 |
| HOXA13 | 1 | 0 |
| HOXD | 1 | 0 |
| HOXD13 | 1 | 1 |
| NIPBL | 0 | 1 |
| PITX2 | 0 | 1 |
| PORCN | 0 | 1 |
| RECQL4 | 0 | 1 |
| SHFM1 | 0 | 1 |
| SHFM2 | 0 | 1 |
| SMC1A | 0 | 1 |
| SMC3 | 0 | 1 |
| SNX3 | 0 | 1 |
| TP63 | 1 | 1 |
